# Proximity to the Promoter and Terminator Regions Regulates the Transcription Enhancement Potential of an Intron

**DOI:** 10.3389/fmolb.2021.712639

**Published:** 2021-07-05

**Authors:** Katherine Dwyer, Neha Agarwal, Alisa Gega, Athar Ansari

**Affiliations:** Department of Biological Science, Wayne State University, Detroit, MI, United States

**Keywords:** transcription, promoter directionality, splicing, intron, gene architecture, gene regulation, gene looping, yeast

## Abstract

An evolutionarily conserved feature of introns is their ability to enhance expression of genes that harbor them. Introns have been shown to regulate gene expression at the transcription and post-transcription level. The general perception is that a promoter-proximal intron is most efficient in enhancing gene expression and the effect diminishes with the increase in distance from the promoter. Here we show that the intron regains its positive influence on gene expression when in proximity to the terminator. We inserted *ACT1* intron into different positions within *IMD4* and *INO1* genes. Transcription Run-On (TRO) analysis revealed that the transcription of both *IMD4* and *INO1* was maximal in constructs with a promoter-proximal intron and decreased with the increase in distance of the intron from the promoter. However, activation was partially restored when the intron was placed close to the terminator. We previously demonstrated that the promoter-proximal intron stimulates transcription by affecting promoter directionality through gene looping-mediated recruitment of termination factors in the vicinity of the promoter region. Here we show that the terminator-proximal intron also enhances promoter directionality and results in compact gene architecture with the promoter and terminator regions in close physical proximity. Furthermore, we show that both the promoter and terminator-proximal introns facilitate assembly or stabilization of the preinitiation complex (PIC) on the promoter. On the basis of these findings, we propose that proximity to both the promoter and the terminator regions affects the transcription regulatory potential of an intron, and the terminator-proximal intron enhances transcription by affecting both the assembly of preinitiation complex and promoter directionality.

## Introduction

One of the conserved features of eukaryotic protein-coding genes that distinguishes them from their prokaryotic counterparts is the presence of non-coding intervening regions called introns. All eukaryotic genes, however, do not contain introns. The proportion of genes containing introns vary from 2.4% in pathogenic ascomycete yeast *Candida glabrata* to 98% in capsular basidiomycete yeast *Cryptococcus neoformans* (Neuvéglise et al., 2011; Goebels et al., 2013). A majority of human (92%) and plant (78%) genes also contain introns (International Human Genome Sequencing Consortium, 2004; Sakharkar et al., 2004; Haas et al., 2005). In budding yeast, a mere 3.8% of genes contain introns, but this small number of genes contributes to nearly 27% of mRNA in exponentially growing yeast cells (Ares et al., 1999; Spingola et al., 1999). Introns substantially enhance the expression of genes that accommodate them (Brinster et al., 1988; Palmiter et al., 1991; Okkema et al., 1993; Duncker et al., 1997; Lugones et al., 1999; Comeron, 2004; Juneau et al., 2006; Charron et al., 2007; Rose, 2008; Shabalina et al., 2010; Gallegos and Rose, 2015; Laxa, 2017; Shaul, 2017; Baier et al., 2020). A number of eukaryotic genes are dependent on introns for their normal expression. Introns influence almost every step of RNA metabolism including transcription, cotranscriptional RNA processing, mRNA decay, mRNA export to cytoplasm and translatability of mRNA (Le Hir et al., 2003; Lu and Cullen, 2003; Carmel and Chorev, 2012; Gallegos and Rose, 2015; Shaul, 2017; Rose, 2019). The molecular basis underlying the intron-mediated regulation, however, is not entirely clear. The ability to enhance gene expression though is not a universal feature of introns.

The role of an intron in enhancing transcription has been conserved during evolution, being exhibited by a diversity of eukaryotic systems including yeast, humans, flies, plants, algae and worms (McKenzie and Brennan, 1996; Juneau et al., 2006; Rose, 2008; Shabalina et al., 2010; Gallegos and Rose, 2015; Laxa, 2017; Shaul, 2017; Baier et al., 2020). Inclusion of just one intron in a transgene has been found to enhance its transcription by many folds (Brinster et al., 1988; Palmiter et al., 1991; Rethmeier et al., 1997; Lacy-Hulbert et al., 2001; Bartlett et al., 2009; Sam et al., 2010). Introns are known to enhance transcription by affecting chromatin structure in the promoter-proximal region and by facilitating the recruitment of general transcription machinery on the promoter. In mammalian cells, an intron facilitates H3K4-trimethylation and H3K9-acetylation in the promoter-proximal regions (Bieberstein et al., 2012). Both of these histone marks help in the recruitment of general transcription machinery to the promoter region. A promoter-proximal intron has also been shown to expedite the recruitment of transcription factors on the promoter region through interaction of U1-snRNP with the components of general transcription machinery (Kwek et al., 2002; Das et al., 2007; Damgaard et al., 2008). In budding yeast, the intron enhances gene expression primarily by affecting transcription and mRNA stability (Furger et al., 2002; Juneau et al., 2006; Parenteau et al., 2008; Parenteau et al., 2011; Moabbi et al., 2012; Agarwal and Ansari, 2016; Petibon et al., 2016). The positive influence of an intron on transcription of several yeast genes has been demonstrated using the nuclear run-on approach (Furger et al., 2002; Moabbi et al., 2012; Agarwal and Ansari, 2016). The intron-mediated enhancement of transcription in yeast requires a splicing-competent intron (Furger et al., 2002; Moabbi et al., 2012; Agarwal and Ansari, 2016). We recently examined the mechanism of intron-mediated enhancement effect in budding yeast and found that a promoter-proximal intron results in the formation of a unique looped gene architecture (Moabbi et al., 2012; Agarwal and Ansari, 2016; Dwyer et al., 2021). We showed that the gene loop facilitated the recruitment of termination factors near the promoter-proximal region, and these termination factors inhibited upstream antisense RNA (uaRNA) synthesis, thereby conferring directionality to the promoter (Agarwal and Ansari, 2016; Al Husini et al., 2020). This results in enhanced transcription of mRNA. In a gene looping-defective mutant, even a splicing-competent intron was unable to enhance transcription, thereby implicating looped gene architecture in intron-mediated transcriptional regulation (Dwyer et al., 2021).

A number of factors influence the intron-mediated enhancement effect. The sequence of an intron is a crucial determinant of its role in regulation of gene expression (Morello et al., 2010; Parra et al., 2011). Another critical factor in determining the regulatory potential of an intron is its proximity to the promoter. It is generally believed that the introns located within the first 1 kbp of the promoter region are most efficient in enhancing gene expression, and the enhancement potential is inversely proportional to the distance of the intron from the promoter element (Callis et al., 1987; Nesic et al., 1993; Jeon et al., 2000; Rose, 2004; Jeong et al., 2006; Rose, 2008; Gallegos and Rose, 2015). There are conflicting reports regarding the ability of a terminator-proximal intron to enhance gene expression (Snowden et al., 1996; Furger et al., 2002; Rose, 2004). A terminator-proximal intron facilitates 3′ end processing of mRNA and termination of transcription (Huang and Gorman, 1990; Nesic et al., 1993; Gunderson et al., 1997; Antoniou et al., 1998; Dye and Proudfoot, 1999; Vagner et al., 2000; McCracken et al., 2002; Awasthi and Alwine, 2003; Kyburz et al., 2006; Millevoi et al., 2006; Kim et al., 2011; Martinson, 2011). This can potentially enhance transcription of the gene. There are, however, a few reports that introns located in the 3′ UTR adversely affect mRNA stability and translatability in mammalian and plant cells (Bourdon et al., 2001; Kertesz et al., 2006; Barrett et al., 2012).

To understand the role of the position of an intron in a gene, we inserted the *ACT1* intron into different positions of *IMD4* and *INO1* genes. Our results show that the proximity to both the promoter and the terminator has a positive influence on transcription of the gene. Like the promoter-proximal intron, the terminator-proximal intron also mediates gene looping and enhances promoter directionality. A terminator-proximal intron though is less efficient than a promoter-proximal intron in enhancing transcription. We further show that both the promoter and terminator-proximal introns facilitate the assembly or stabilization of the PIC on the promoter. Our findings reveal that an intron enhances gene expression by influencing multiple aspects of promoter function and suggest that it accomplishes this by modulating the architecture of actively transcribing genes. We propose that proximity to both the promoter and terminator is crucial for the transcription enhancement potential of an intron.

## Materials and Methods

### Yeast Strains

Yeast strains used in this study are listed in Supplementary Table S1.

### Cell Cultures

Cultures were started by inoculating 5 ml of YP-dextrose medium with colonies from a freshly streaked plate and grown at 30°C with gentle shaking overnight. Next morning, cultures were diluted (1:100) to appropriate volume and grown to A_600_ ∼0.4–0.6. Equal number of cells were used for strand-specific RT-PCR, 3C, ChIP or strand-specific TRO assays.

### Chromosome Conformation Capture Assay

**FIGURE 1:**
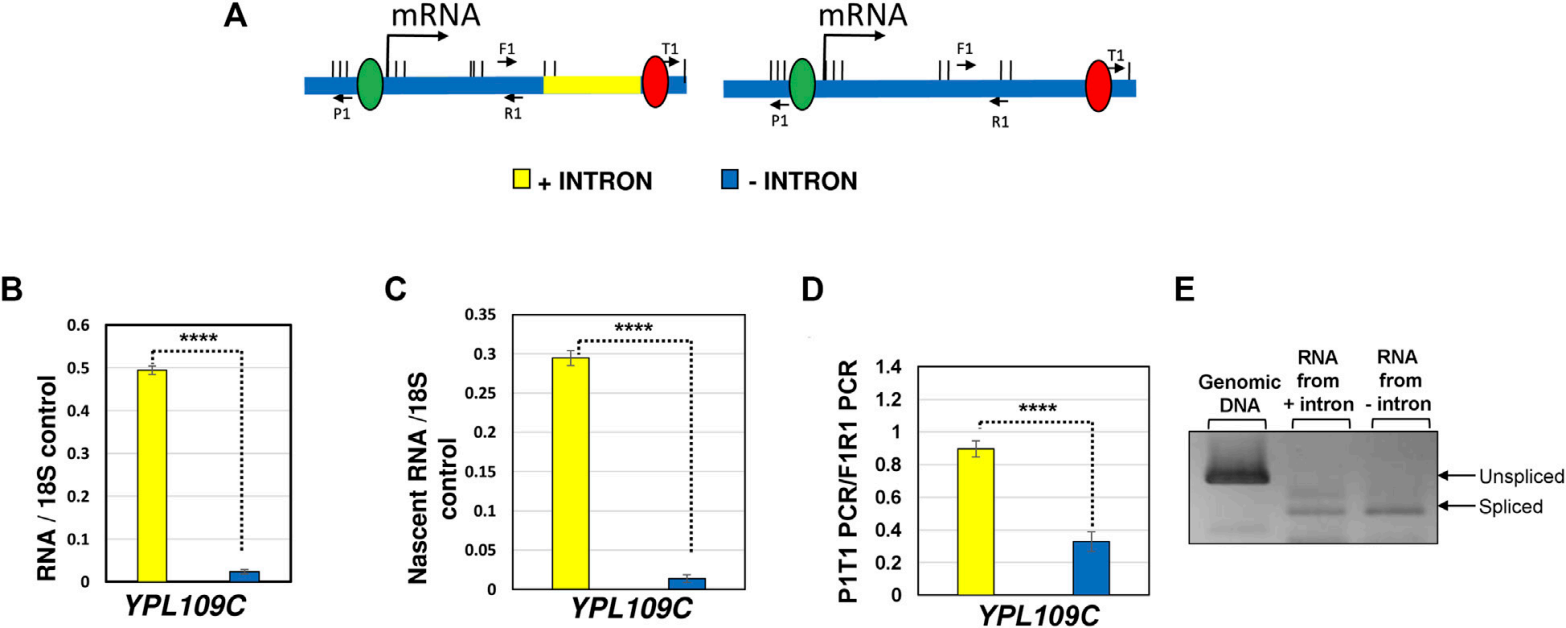
Deletion of the terminator-proximal intron of *YPL109C* reduces both the transcription and looped conformation of the gene. **(A)** Schematic depiction of *YPL109C* with the intron present (yellow) and intron deleted (blue) indicating the position of primers used for RT-PCR, TRO and 3C analyses. **(B)** Quantification of total RNA level of *YPL109C* measured by RT-PCR approach in the presence of the terminator-proximal intron (yellow bar) and the absence of intron (blue bar). The 18S RNA signal was used as a normalization control. **(C)** Quantification of nascent RNA level of *YPL109C* measured by TRO approach in the presence of the terminator-proximal intron (yellow bar) and the absence of intron (blue bar). The 18S RNA signal was used as a normalization control. **(D)** Gene looping of *YPL109C* measured in terms of P1T1 PCR signal by 3C approach in the presence (yellow bar) and absence (blue bar) of an intron. The F1R1 PCR represents the loading control that was used to ensure equal amounts of template DNA were present in each 3C PCR reaction. The values and error bars for each condition indicate the mean ± standard deviation. Statistical significance (*p*-values) was determined using the two-tailed paired Student’s *t*-test. Four asterisks (****) indicate *p* value smaller than 0.0001 (*p* ≤ 0.0001). **(E)** RT-PCR analysis of *YPL109C* mRNA in the construct with intron (+ intron) and without intron (− intron). Genomic DNA PCR of intron-containing strain indicates the position of unspliced transcripts. The expected positions of spliced and unspliced transcripts obtained by RT-PCR approach for each construct is indicated.

**FIGURE 2:**
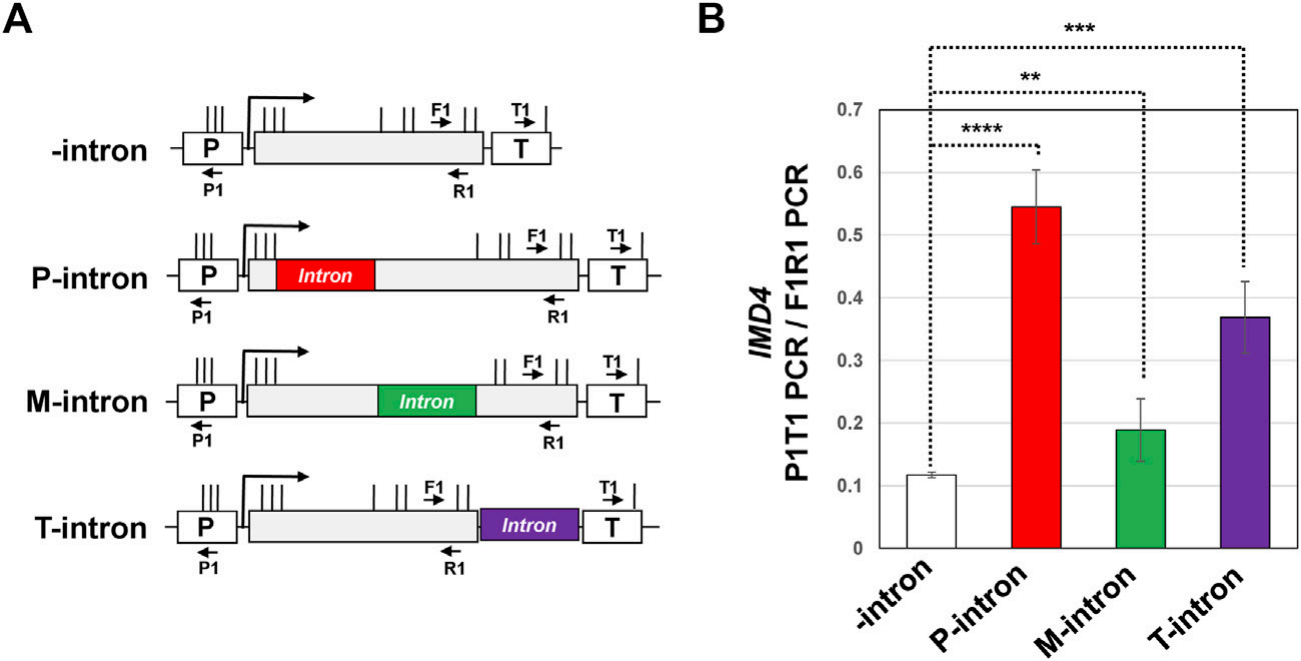
Position of an intron within the gene impacts gene looping of *IMD4*. **(A)** Schematic depiction of *IMD4* gene without intron (-intron, white), and with *ACT1* intron inserted near the promoter (P-intron, red), in the middle of the gene (M-intron, green) and in the proximity of the terminator (T-intron, purple) region. “p” and “T” stand for the promoter and terminator respectively. Vertical lines show position of restriction cut sites, while P1, T1, F1 and R1 are the position of primers used to amplify 3C PCR products. **(B)** 3C analysis of *IMD4* in the absence of an intron (white bar), and with the intron inserted near the promoter (red bar), in the middle (green bar) and toward the terminator region of the gene (purple bar). P1T1 PCR represents gene looping signal, while F1R1 PCR represents the loading control that was used to ensure equal amounts of template DNA were present in each 3C PCR reaction. Results presented here represent three biological replicates and six technical replicates. The values and error bars for each condition indicate the mean ± standard deviation. Statistical significance (*p*-values) was determined using the two-tailed paired Student’s *t*-test. Two asterisks (**) signify a *p* value equal to or smaller than 0.01 (*p* ≤ 0.01). Three asterisks (***) indicate *p* value equal/smaller than 0.001 (*p* ≤ 0.001). Four asterisks (****) indicate *p* value equal/smaller than 0.0001 (*p* ≤ 0.0001). Any non-significant difference is represented by ns (*p* > 0.05).

3C experiments were performed as described previously in (El Kaderi et al., 2012). The primers used for 3C analysis are shown in Supplementary Table S2. The restriction enzymes used for chromatin digestion of *IMD4* gene were Alu1 and Dra1. Each experiment was performed with at least four independently grown cultures. The P1T1 PCR signals obtained using P1 and T1 primers flanking the promoter and terminator regions respectively were normalized with respect to F1-R1 PCR signal that amplifies an uncut region within the gene as shown in Figures 1D, 2B. All controls described in El Kaderi et al. (2012) were performed in 3C experiments. These included no-formaldehyde-crosslinking control, no-ligation control and primer efficiency control.

### Chromatin Immunoprecipitation

ChIP experiments (crosslinking, cell lysis and isolation of chromatin) were performed as described previously (El Kaderi et al., 2009). Different strains were constructed by tagging TFIIB and the Ccl1 subunit of TFIIH as indicated in Supplementary Table S1. Anti-HA-Agarose beads, used to pull down HA-tagged subunits, were obtained from Sigma. IgG-Sepharose beads, purchased from GE Healthcare, were used to pull down TAP-tagged Ccl1 subunit of TFIIH. For ChIP analysis, primers used for ChIP PCR are shown in Supplementary Table S2. Each experiment was repeated with at least four independently grown cultures. Background ChIP signal for each TFIIB and TFIIH experiment was determined using no tag control version of strains. All TFIIB and TFIIH ChIP signals were first normalized with no tag control, and then with input.

### Transcription Analysis

Transcription analysis was performed by RT-PCR approach as described previously (El Kaderi et al., 2009). The primers used for RT-PCR analysis are shown in Supplementary Table S2.

### Strand-Specific “Transcription Run-On” Assay

The strand-specific “Transcription Run-On” (TRO) assay was performed as described in (Dhoondia et al., 2017). The primers used for making cDNA and PCR are described in Supplementary Table S2.

### Quantification

The data shown in each figure is the result of at least three biological replicates and six technical replicates. The quantification and statistical analysis were performed as described in (El Kaderi et al., 2012). Error bars represent one unit of standard deviation. *p*-values were calculated by two-tailed student t-test (Student, 1908).

## Results

### A Terminator-Proximal Intron Enhances Transcription of Yeast Genes

A vast majority of intron-containing genes in budding yeast have an intron in the promoter-proximal position. A small number of yeast genes carry an intron in the middle of the gene, and an even fewer number have them in the vicinity of the terminator region. It has been shown that a promoter-proximal intron enhances transcription of a gene by facilitating the recruitment of general transcription machinery and by enhancing transcription directionality (Kwek et al., 2002; Das et al., 2007; Damgaard et al., 2008; Bieberstein et al., 2012; Agarwal and Ansari, 2016). The effect of a promoter-proximal intron on transcription has been conserved during evolution as it is exhibited by simple eukaryotes like budding yeast as well as the most complex mammalian systems. There is, however, no concrete evidence regarding the transcription enhancement potential of a terminator-proximal intron. *YPL109C* is one of the few yeast genes that contain a terminator-proximal intron. We deleted the terminator-proximal intron of *YPL109C*, and measured transcription of the gene in the presence and absence of the intron (Figure 1A). Steady-state RNA analysis revealed that *YPL109C* mRNA level decreased by about 10-folds in the absence of the intron (Figure 1B). Transcription Run-On (TRO) analysis, which measured the transcription of nascent mRNA, revealed similar results (Figure 1C). These results demonstrate that even a terminator-proximal intron has the potential to enhance the transcription of the gene.

**FIGURE 3:**
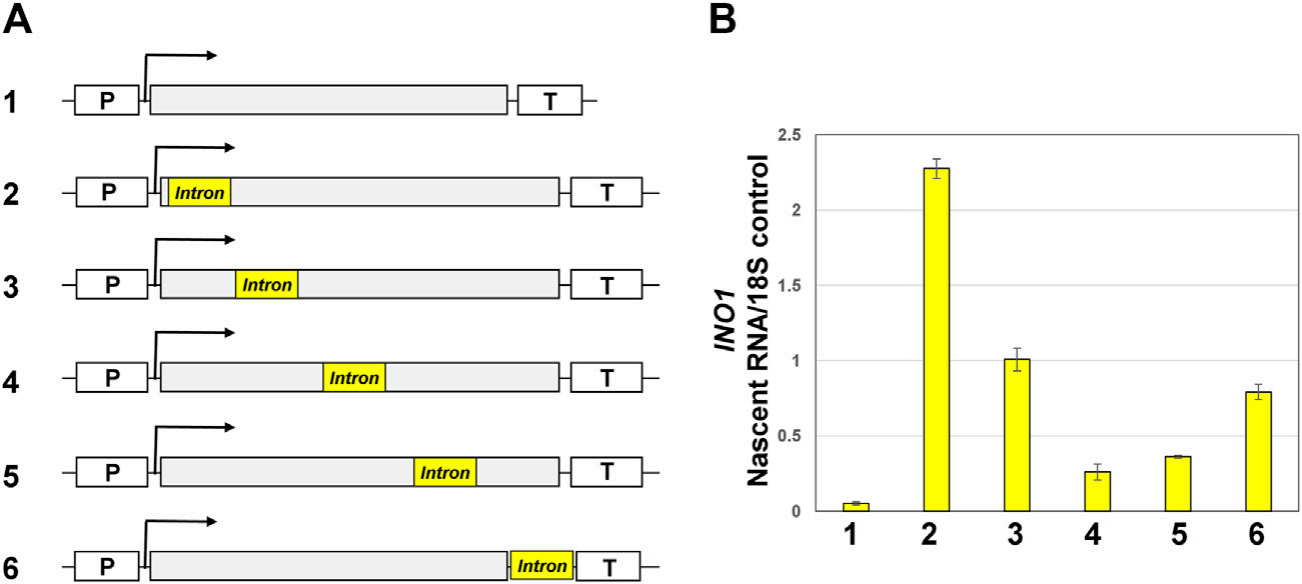
Position of the intron in *INO1* gene influences the transcription of the gene. **(A)** Schematic representation of *INO1* gene without intron (1) and with the *ACT1* intron inserted at five different positions along the body of the gene (2–6). “p” and “T” stand for the promoter and terminator respectively. **(B)** Quantification of TRO data of *INO1* gene in the absence of an intron (#1) and the presence of an intron at 100 bp position (#2), 500 bp (#3), 800 bp (#4), 1,400 bp (#5), and 10 bp downstream of (TGA + 10) (#6). The transcript levels of 18S were used as the normalization control.

To better understand the role of the position of an intron in a gene on its transcription regulatory potential, it required inserting the same intron sequence at different positions of a gene and measuring transcription of the gene at every position. We have earlier showed that *ACT1* intron enhances transcription of *INO1* in an activator-independent manner (Moabbi et al., 2012). Furthermore, we demonstrated that transcription activation potential of *ACT1* intron was not due to the presence of a promoter or enhancer sequence element within the intron but was strictly dependent on the splicing potential of the intron. We therefore inserted *ACT1* intron into five different positions of *INO1* gene following the strategy described in Moabbi et al. (2012). *INO1* is an intron-less gene, which is induced in the absence of inositol in the growth medium. We have previously demonstrated enhancement of *INO1* transcription under non-inducing conditions upon insertion of an intron near the promoter region of the gene (Moabbi et al., 2012). Here we inserted *ACT1* intron at four additional positions of *INO1* gene as shown in Figure 3A. The intron was inserted in the 1,602 bp long *INO1* gene at 100, 500, 800 and 1,400 bp positions in the coding region of the gene, and one 10 bp downstream of the coding region in the 3′ UTR of the gene. At each position of the intron, transcription was monitored by strand-specific TRO approach as described in Medler and Ansari (2015). TRO assay measures nascent mRNA, which is not affected by half-life of mRNA, and accurately reflects transcription of the gene. In the absence of an intron, there is very low level basal transcription of the gene. Insertion of the intron at 100 bp position enhanced nascent mRNA levels by about 43 times over the intronless version (Figure 3B). At the 500 bp position, there was only 20 times stimulation in transcript level by the intron over the intron-less control. Thus, the enhancement effect of an intron at the 500 bp position registered a decline by about 50% compared to the 100 bp position (Figure 3B). Introns inserted at 800 and 1,400 bp position resulted in a mere 6 times and 8 times increase respectively in transcription over the intron-less control. These results are in agreement with the published results from other organisms as they clearly demonstrate that the enhancement effect of the intron on gene expression decreases with the increase in distance of the intron from the promoter region (Callis et al., 1987; Nesic et al., 1993; Rose, 2004). There was, however, a surprise when we inserted the intron at a position 10 bp downstream of the coding region. The 3′ UTR intron enhanced *INO1* transcription by about 17 times (Figure 3B). These results suggest that the proximity to the terminator region partially restores the enhancement potential of an intron on gene expression. Yet, the terminator-proximal intron was not as efficient as the promoter-proximal intron in enhancing transcription of *INO1*. Although the promoter-proximal intron was spliced with slightly lower efficiency, overall introns were spliced with similar efficiency at each of these five positions (Supplementary Figure S1), thereby suggesting that the observed results were not because of the differential splicing efficacy of introns.

**FIGURE 4:**
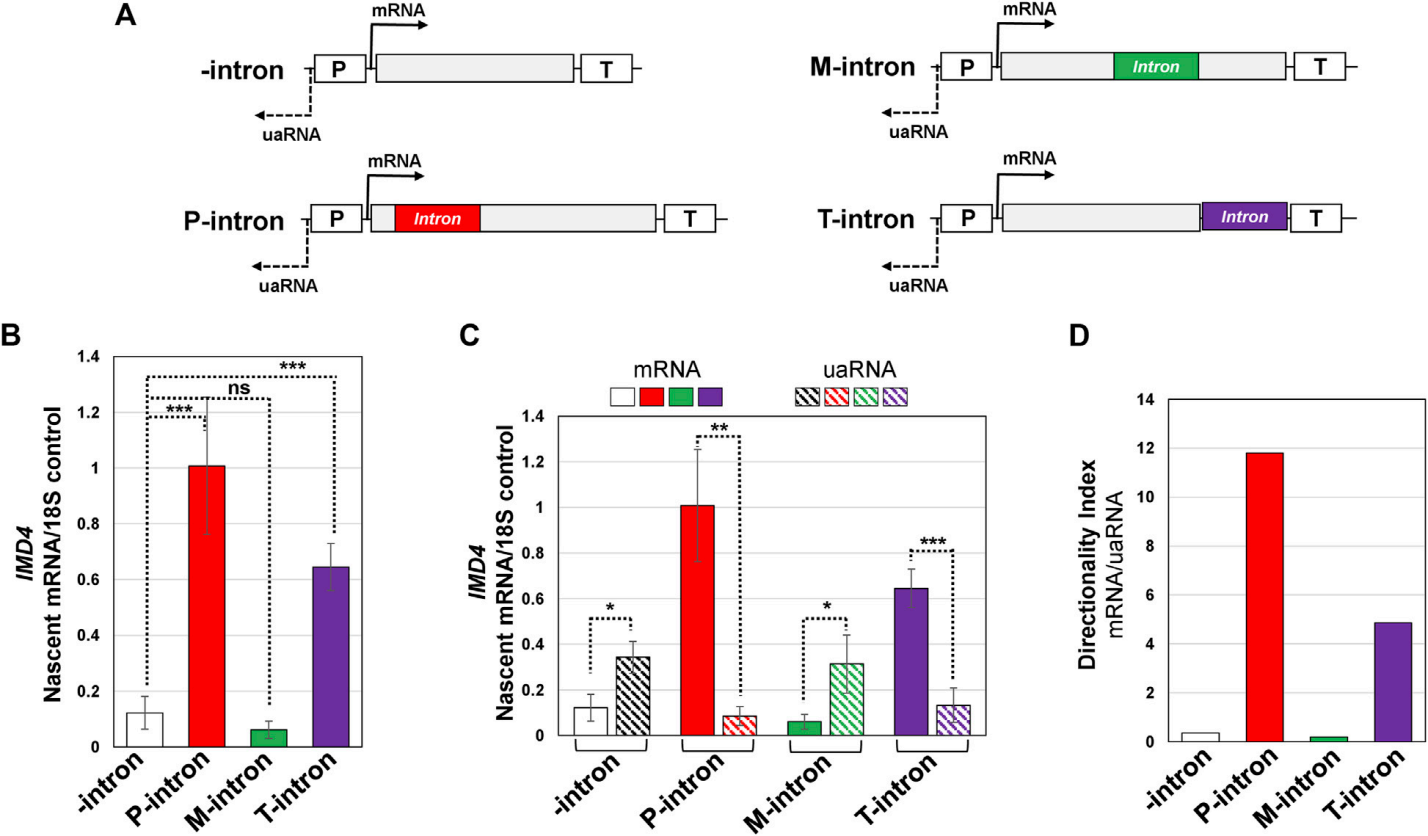
Both the promoter and terminator proximal introns enhance transcription and promoter directionality of *IMD4* gene. **(A)** Schematic depiction of *IMD4* gene without intron (-intron, white), and with *ACT1* intron inserted near the promoter (P-intron, red), in the middle of the gene (M-intron, green) and in the proximity of the terminator (T-intron, purple) region. “p” and “T” stand for the promoter and terminator respectively. **(B)** TRO analysis of *IMD4* in the absence of an intron (white bar), and with the intron inserted near the promoter (red bar), in the middle (green bar) and toward the terminator region of the gene (purple bar). **(C)** Measurement of mRNA and uaRNA levels of *IMD4* in the 400 bp upstream (uaRNA) and downstream (mRNA) promoter proximal region by strand-specific TRO approach in the absence of an intron (white bar for mRNA, diagonal black pattern bar for uaRNA), and with the intron inserted near the promoter (red bar for mRNA, diagonal red pattern bar for uaRNA), in the middle (green bar for mRNA, diagonal green pattern bar for uaRNA) and toward the terminator region of the gene (purple bar for mRNA, diagonal purple pattern bar for uaRNA). **(D)** Directionality index of *IMD4* without intron (white bar) and with the intron present at three different positions (red, green and purple bars) by comparing levels of mRNA vs. uaRNA produced. The 18S signal was used as a normalization control in these experiments. Results presented here represent three biological replicates and six technical replicates. The values and error bars for each condition indicate the mean ± standard deviation. Statistical significance (*p*-values) was determined using the two-tailed paired Student’s *t*-test. One asterisk (*) signifies a *p* value equal to or smaller than 0.05 (*p* ≤ 0.05). Any non-significant difference is represented by ns (*p* > 0.05).

The *INO1* gene does not contain a natural intron. The results obtained with *INO1* therefore need to be substantiated with a gene containing a natural intron. We, therefore, repeated the experiment with *IMD4*, which is a natural intron-containing gene. We first constructed a strain containing the intron-less version of *IMD4* gene as described in Moabbi et al. (2012). We then inserted *ACT1* intron at three different positions of the *IMD4* gene; one at 461 bp position (promoter-proximal), a second one at 750 bp position within the coding region (middle), and a third one in 3′ UTR at the position 20 bp downstream of the coding region (terminator-proximal) as shown in Figure 4A. Like *INO1*, we have previously demonstrated that *ACT1* intron enhances transcription of *IMD4* in a splicing-dependent manner as mutation of either 5′ or 3′ splice sites completely abolished transcription enhancement potential of the intron (Agarwal and Ansari, 2016). Transcription of the gene was then monitored by strand-specific TRO approach as described earlier. The results show very little detectable transcription in the absence of an intron (Figure 4B). Insertion of the intron at 461 position (promoter-proximal) stimulated transcription of the gene by about 10-fold over the intronless version. The 750 intron (middle) did not result in enhancement of transcription over the intron-less control as the TRO signal obtained was similar to that in the absence of an intron. In the presence of a terminator-proximal intron, however, TRO signal registered an increase of almost six-fold over the intron-less control (Figure 4B). The enhancement effect of terminator-proximal intron is statistically significant as indicated by the *p*-value of 2.6E-04 (Figure 4B). Although the promoter-proximal intron was spliced with slightly lower efficiency, overall introns were spliced with similar efficiency at each of these three positions (Supplementary Figure S2), thereby suggesting that the observed results were not because of the differential splicing behavior of introns. These results clearly demonstrate that an intron regains its transcription enhancement potential with increasing proximity to the terminator region of the gene in budding yeast. A logical conclusion of these results is that the proximity to both the promoter and terminator regions contributes to the enhancement potential of an intron on gene expression.

### Terminator-Proximal Intron Affects Gene Looping and Promoter Directionality

We have recently demonstrated a novel role of a promoter-proximal intron in enhancing transcription by conferring directionality to the promoter-bound polymerase to transcribe in the sense direction and inhibit transcription in the upstream anti-sense direction (Agarwal and Ansari, 2016). To determine if the transcription regulatory function of a terminator-proximal intron is also due to its effect on transcription directionality, we performed TRO in the promoter-proximal 800 bp window; 400 bp downstream (mRNA) and upstream (uaRNA) of the promoter as shown in Figure 4A. The terminator-proximal intron stimulated transcription in the promoter-proximal sense direction (mRNA) by about six fold (Figure 4B, solid purple bar), while transcription in the upstream anti-sense direction (uaRNA) decreased by about 2.5 fold over the intron-less control (Figure 4C, diagonal purple pattern bar). The promoter-proximal intron had a similar effect on mRNA and uaRNA levels as expected (Figure 4C, solid red and diagonal red pattern bar). The middle intron, which did not enhance transcription of the gene (Figure 4C, solid green bar), also had no significant effect on uaRNA transcription over the intron-less control (Figure 4C, diagonal green pattern bar).

To better understand the role of the position of an intron on promoter directionality, we calculated the directionality index by dividing nascent mRNA levels with nascent uaRNA levels for the intron inserted at different positions of the *IMD4* gene. The directionality index in the presence of a promoter-proximal intron increased by about 24 fold (Figure 4D, red bar), and in the presence of a terminator-proximal intron by about 10 fold (Figure 4D, purple bar) over the intron-less control. In contrast, the middle intron did not affect the directionality index over the intron-less version of the gene (Figure 4D, green bar). Thus, a terminator-proximal intron also enhances transcription directionality in a manner similar to that observed by the promoter-proximal intron.

Our published results demonstrated that the intron-dependent promoter directionality was through gene looping, which is the interaction of the promoter and terminator regions of a gene in a transcription-dependent manner (Agarwal and Ansari, 2016; Al Husini et al., 2020). Juxtaposition of the terminator and promoter regions places termination factors in the vicinity of the promoter region leading to termination of uaRNA transcription. We therefore examined if a terminator-proximal intron also confers looped gene architecture. Gene looping was detected by the “Chromosome Conformation Capture” (3C) approach. This procedure converts the promoter-terminator interaction into quantitatively measurable PCR products obtained using the primer pair flanking the promoter and terminator region, as schematically represented in Figure 2A. 3C analysis was performed with the intron inserted at different positions of the *IMD4* gene following the protocol described in (El Kaderi et al., 2012). In all 3C experiments, complete digestion of chromatin between promoter and terminator, crosslinking-dependence and ligation-dependence of the results was routinely monitored. All 3C PCR products were verified by sequencing. Using our modified high resolution 3C approach, we have previously demonstrated a specific interaction of promoter and terminator regions of *INO1* in a transcription-dependent manner (El Kaderi et al., 2009; Moabbi et al., 2012). As expected, the 3C assay detected a compact gene architecture with the promoter and terminator in close proximity for the construct with a promoter-proximal intron (Figure 2B, red bar). The promoter-terminator proximity measured in terms of P1T1 PCR signal was five fold more in the construct with the promoter-proximal intron compared to that in the absence of an intron. In the construct with the intron in the middle of the gene, gene architecture was comparable to that in the absence of the intron (Figure 2B, green bar). In the presence of the terminator-proximal intron, however, the promoter-terminator proximity was partially restored as P1T1 PCR signal registered a 3.6 fold increase over the intron-less strain (Figure 2B, purple bar). *YPL109C* gene, which has a terminator-proximal intron, similarly exhibited an intron-dependent change in gene architecture resulting in the promoter coming in proximity of the terminator of the gene (Figure 1D).

### Terminator-Proximal Intron Also Affects PIC

**FIGURE 5:**
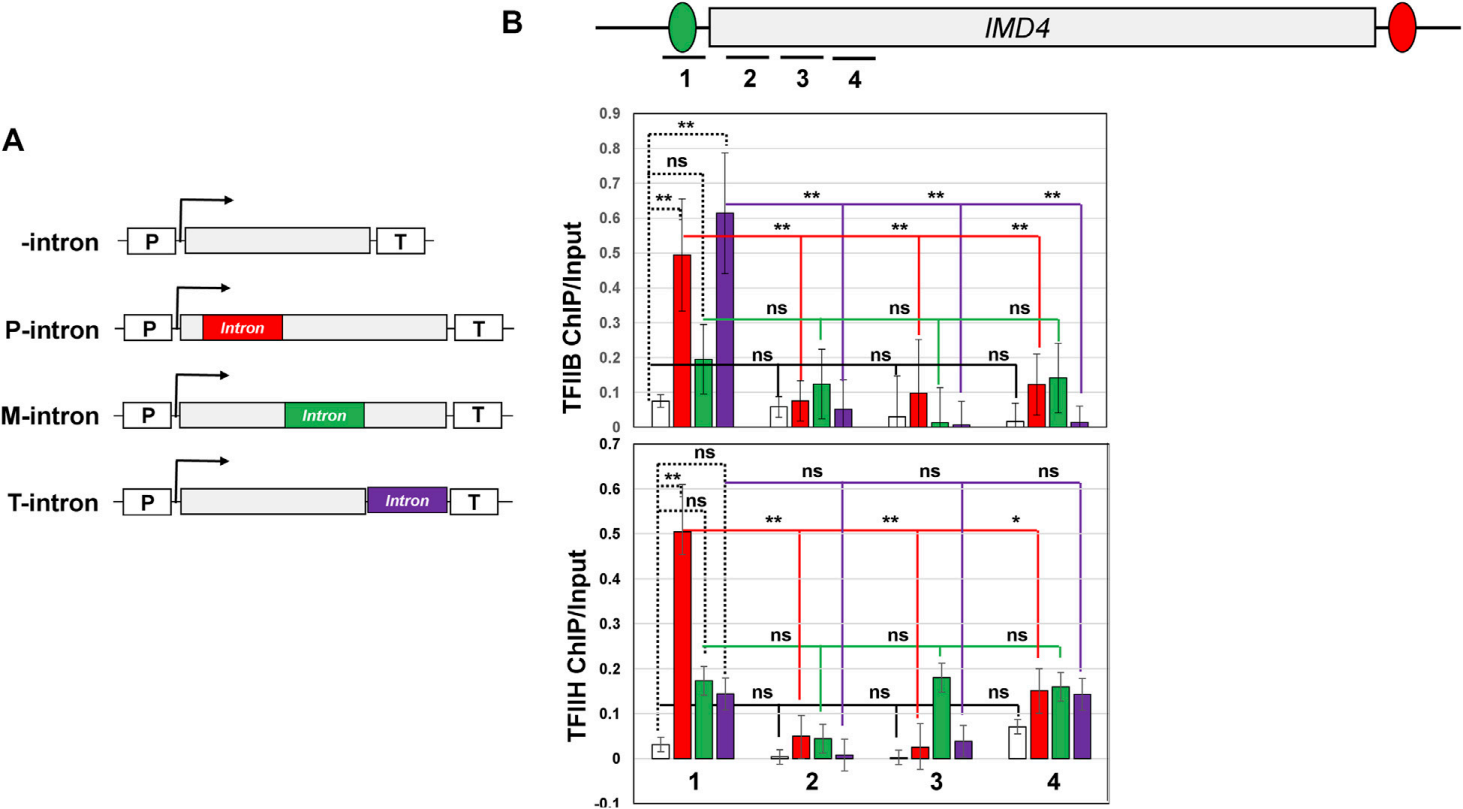
Position of intron affects TFIIB and TFIIH promoter occupancy for *IMD4* gene. **(A)** Schematic depiction of *IMD4* without intron and with the *ACT1* intron inserted near the promoter (P-intron), in the middle (M-intron) and near the terminator region (T-intron) of the gene. “p” and “T” stand for the promoter and terminator respectively. **(B)** Schematic depiction of *IMD4* gene with intron indicating the position of primer pairs used in ChIP analysis. **(C)** ChIP analysis showing crosslinking of HA-tagged TFIIB to the promoter and downstream regions of *IMD4* with promoter-proximal intron (red bar), middle intron (green bar), terminator-proximal intron (purple bar) and without intron (white bar). **(D)** ChIP analysis showing crosslinking of TAP-tagged Ccl1 subunit of TFIIH to the promoter and downstream coding regions of *IMD4* with promoter-proximal intron (red bar), middle intron (green bar), terminator-proximal intron (purple bar) and without intron (white bar). The Input signal, representing DNA prior to immunoprecipitation, was used as normalization control. ChIP results presented here represent three biological replicates and six technical replicates. The values and error bars for each condition indicate the mean ± standard deviation. Statistical significance (*p*-values) was determined using the two-tailed paired Student’s *t*-test. One asterisk (*) signifies a *p* value equal to or smaller than 0.05 (*p* ≤ 0.05). Two asterisks (**) signify a *p* value equal to or smaller than 0.01 (*p* ≤ 0.01). Any non-significant difference is represented by ns (*p* > 0.05).

In the mammalian system, a promoter proximal intron has been shown to enhance transcription by facilitating the recruitment or stabilization of the recruited preinitiation complex (PIC) on the promoter (Kwek et al., 2002; Damgaard et al., 2008; Bieberstein et al., 2012). Whether the intron plays a similar role in the recruitment/stabilization of PIC in yeast, however, has never been investigated. Therefore, to determine if the intron facilitates assembly/stabilization of PIC in yeast, we monitored the occupancy of two GTFs, TFIIB and TFIIH, on the promoter of *IMD4* in the presence and absence of an intron. We monitored the occupancy of GTFs in a strain that has HA-tagged TFIIB and TAP-tagged cyclin subunit Ccl1 of TFIIH. ChIP was performed to look directly at the occupancy of two GTFs at the promoter and within the 300 bp promoter downstream coding region. Our results show that the promoter occupancy of TFIIB was enhanced by about five-fold in the presence of a promoter-proximal intron over the intron-less control (*p*-value of 0.01) (Figure 5B, upper panel, red bar). Some signal for TFIIB is detected in the 300 bp promoter downstream region, but the signal is 70–80% less compared to that on the promoter. A comparison of *p*-values shows that the decrease in TFIIB signal in the promoter-proximal coding region relative to the promoter is statistically significant (Figure 5B, upper panel red bars). Similar results were obtained for Ccl1. The Ccl1 ChIP signal registered a ten-fold increase in the presence of the promoter-proximal intron over no-intron control (*p*-value of 0.01) (Figure 5B, lower panel, red bar). Like TFIIB, Ccl1 signal also registered a 70–80% decline in the 300 bp promoter-downstream coding region (Figure 5B, lower panel, red bars). These results clearly show that, like in mammalian cells, an intron facilitates the assembly or stabilization of PIC in yeast as well.

Since we have shown that the position of an intron in a gene affects promoter directionality, we next examined if the position of the intron also affects its ability to recruit or stabilize binding of PIC components on the promoter region. We therefore checked occupancy of TFIIB and TFIIH subunit Ccl1 in the presence of the intron in the middle region and the terminator-proximal region of *IMD4* as shown in Figure 5A. In the presence of a middle intron, TFIIB ChIP-signal in the promoter region increased by about two fold (Figure 5B, upper panel, green bar), while Ccl1 signal registered a nearly three fold increase (Figure 5B, lower panel, green bar) over the intron-less control. Although there was a small increase in the transcription factors occupancy, it was statistically not significant as indicated by the *p*-values (*p*-values 0.67 for TFIIB and 0.08 for Ccl1). Thus, unlike the promoter-proximal intron, the middle intron does not have a significant effect on the promoter occupancy of the general transcription factors. These results are in accordance with the effect of middle intron on transcription and directionality.

The terminator-proximal intron, like the promoter-proximal intron, though has a positive impact on PIC occupancy. The TFIIB promoter ChIP signal increased by about six fold in the presence of the terminator-proximal intron over the intron-less version (*p*-value 0.005) (Figure 5B, upper panel, purple bar). TFIIH promoter ChIP signal also increased by around three fold in the presence of the terminator-proximal intron, but the increase in signal is statistically not significant (*p*-value 0.22) (Figure 5B, lower panel, purple bar). These results are corroborated by the TFIIB and TFIIH ChIP-signals in the promoter-proximal coding region. TFIIB ChIP signal exhibits a statistically significant decline in the coding region compared to the promoter (Figure 5B, upper panel), while TFIIH signal does not exhibit a statistically significant change in the coding region relative to the promoter (Figure 5B, lower panel).

These results suggest that a promoter-proximal intron does indeed enhance occupancy of transcription factors at the promoter, while a terminator-proximal intron may only help with occupancy of certain factors of the PIC such as TFIIB. This may also explain why the terminator-proximal intron is not as efficient as the promoter-proximal intron in enhancing transcription.

### Effect of Position of Intron on Termination of Transcription

**FIGURE 6:**
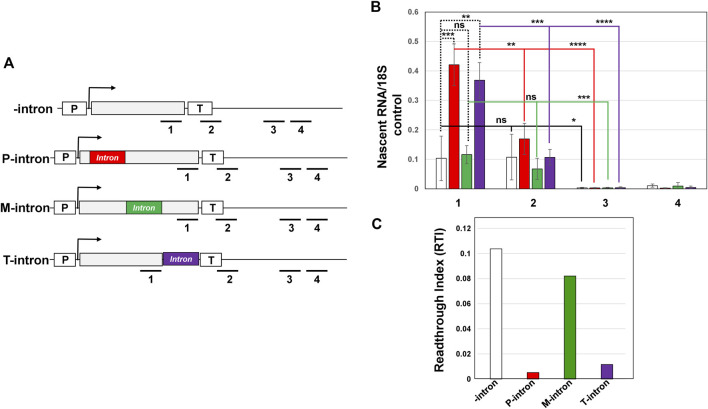
Presence of an intron does not affect termination of transcription of *IMD4*. **(A)** Schematic depiction of *IMD4* with intron inserted near the promoter (P-intron), middle of the gene (M-intron) and near the terminator region (T-intron), showing the position of primers 1, 2, 3, and four used in strand-specific TRO analysis. “p” and “T” stand for the promoter and terminator respectively. **(B)** Strand-specific TRO analysis showing that the polymerase did not readthrough in the regions 2, 3, and four downstream of the terminator signal of *IMD4* in the construct P-intron (red bar), M-intron (green bar) and T-intron (purple bar). The 18S signal was used as a normalization control in these experiments. Results presented here represent three biological replicates and six technical replicates. The values and error bars for each condition indicate the mean ± standard deviation. Statistical significance (*p*-values) was determined using the two-tailed paired Student’s *t*-test. One asterisk (*) signifies a *p* value equal to or smaller than 0.05 (*p* ≤ 0.05). Two asterisks (**) signify a *p* value equal to or smaller than 0.01 (*p* ≤ 0.01). Three asterisks (***) indicate *p* value equal/smaller than 0.001 (*p* ≤ 0.001). Four asterisks (****) indicate *p* value equal/smaller than 0.0001 (*p* ≤ 0.0001). Any non-significant difference is represented by ns (*p* > 0.05). **(C)** Readthrough Index (RTI) was calculated by dividing the TRO signal intensity beyond the 3′ end of the gene (region 4) with the signal value before the 3′ end within the coding region (region 1).

Since a terminator proximal intron has been shown to affect termination in metazoans, and intron-dependent gene looping involves a terminator-intron interaction as well, we next examined if the presence of an intron in general, and that of the terminator-proximal intron in particular affect termination of transcription. We monitored termination by transcription run-on (TRO) assay as described in Dhoondia et al. (2017) for *IMD4* gene with the intron inserted at three different positions as shown in Figure 6A. TRO assay detects the presence of transcriptionally active polymerases on a gene as shown in Figure 6B. To determine the role of the position of an intron on termination of transcription, we calculated the readthrough index (RTI) in constructs with the intron at different positions in the gene. RTI was calculated by dividing the TRO signal intensity beyond the 3′ end of the gene with the signal value before the 3′ end within the coding region. A termination defect results in a higher RTI value, while efficient termination yields a low RTI value. In the presence of an intron in the promoter-proximal position and terminator-proximal position, the RTI value registered a 20-fold and 10-fold decrease respectively over the intronless control (Figure 6C, red bar and purple bar). In the presence of an intron in the middle position, however, the RTI value decreased by a mere 1.2 fold over that in the absence of an intron (Figure 6C, green bar). These results suggest that the position of an intron affects termination of transcription. Both the promoter-proximal and terminator-proximal introns result in efficient termination, while intronless and middle intron versions exhibited inefficient termination. In general, TRO readthrough signal beyond the 3′ end of gene is expected to be higher in the termination defective mutants than in wild type cells (Birse et al., 1998). This was, however, not the case in constructs without an intron and with an intron in the middle position. The TRO readthrough signals in the presence of an intron at all three positions as well as in the absence of intron were very similar (Figure 6B). Whether the position of an intron in yeast affects termination, therefore, needs more scrutiny.

## Discussion

A comparison of the active RNAPII molecules present on naturally intron-less and intron-containing genes was done by nuclear run on, enabling us to gauge the direct effect of introns on transcription. RNAPII density was almost doubled on intron containing genes and the transcription rate of intron-containing genes was 2.5 times more than that of intron-less genes (Pelechano et al., 2010). This indicates that intron containing genes of yeast are highly transcribed, in accord with their outsized contribution to cellular mRNA. Strand-specific TRO analysis, which directly measures transcription, also revealed that a number of yeast genes exhibit higher transcription with their natural intron (Furger et al., 2002; Moabbi et al., 2012; Agarwal and Ansari, 2016). Most of these genes contained a promoter-proximal intron, known to facilitate the PIC assembly on the promoter. The effect of a terminator-proximal intron on gene transcription has not been thoroughly investigated in yeast or higher eukaryotes. Whether a terminator-proximal intron affects PIC assembly is also not known. There are, however, a few reports that suggest a direct role of a terminator-proximal intron in 3′ end processing of mRNA (Dye and Proudfoot, 1999; Vagner et al., 2000; McCracken et al., 2002). Whether this has any impact on overall transcription was not clear from these studies.

Studies with cultured mammalian cells have found that the 3′ splice-site of the terminator-proximal intron had an adverse effect on trimethylation of H3K36, a chromatin mark normally associated with transcription elongation that must be removed to facilitate termination (Kim et al., 2011). A mutation in the 3′ splice-site of a terminator-proximal intron in the beta-globin gene caused enrichment of this mark in the 3′ region of the gene, which is expected to inhibit termination leading to an overall lowering of transcription. Multiple studies have pointed out the positive influence of terminator-proximal introns on mRNA 3′ end processing (Niwa et al., 1990; Nesic et al., 1993; Dye and Proudfoot, 1999; Vagner et al., 2000; McCracken et al., 2002). In line with our published work wherein we demonstrated that the promoter-proximal intron enhances transcription of both *INO1* and *IMD4* in a splicing-dependent manner (Moabbi et al., 2012; Agarwal and Ansari, 2016), we thought it logical to show that the terminator-proximal intron also enhances transcription in a splicing-dependent fashion. We were, however, unable to insert either a 5′ splice site or 3′ splice site mutated intron near the terminator region of either *INO1* or *IMD4*. The strains with the mutated intron near the terminator region were not viable. Instead we show here that the terminator-proximal intron of both *INO1* or *IMD4* is spliced efficiently (Supplementary Figures S1, S2).

In budding yeast, most of the intronic genes contain a single intron located in the vicinity of the promoter. The contribution of the position of an intron, especially the terminator-linked intron, on gene expression therefore remained largely unexplored. This is the first systematic study analyzing the role of the position of an intron within a gene on its transcription in budding yeast. Our results reaffirm the view emerging from studies with higher eukaryotes that the position of an intron within a gene has a strong bearing on its transcription. Unlike in higher eukaryotes, however, we found that the terminator-linked intron in yeast affects promoter transcription in a manner similar to the promoter-proximal intron. The terminator intron inhibits uaRNA transcription in the vicinity of the promoter region, thereby enhancing promoter directionality. The terminator intron also affects the occupancy of TFIIB on the promoter in a manner similar to a promoter-proximal intron. The occupancy of TFIIH, a general transcription factor that is recruited last during assembly of PIC, however, is not significantly enhanced by the terminal intron. This may explain why a terminator-linked intron is not as efficient in enhancing transcription as the promoter-proximal intron. Unlike in budding yeast, higher eukaryotes contain multiple introns distributed all over the genes. This study will serve as a paradigm for investigating the role of terminator-proximal introns on gene expression in higher eukaryotes.

## Data Availability

The raw data supporting the conclusion of this article will be made available by the authors, without undue reservation.
