# Supplementary material for: Proximity to the Promoter and Terminator Regions Regulates the Transcription Enhancement Potential of an Intron
Source: Front Mol Biosci. 2021 Jul 5;8:712639. doi: 10.3389/fmolb.2021.712639 (PMC8287100; doi:10.3389/fmolb.2021.712639)
Supplement: Supplementary file 1 [file DataSheet1.PDF]

# *Supplementary Material*

**Table S1: Yeast Strains**

| Strain | Genotype | Reference |
|---|---|---|
| BY4733 | *MATa his3Δ200 trp1Δ63 leu2Δ0 met15Δ0 ura3Δ0* | |
| AA6 | *MATa his3Δ200 trp1Δ63 leu2Δ0 met15Δ0 ura3Δ0 HPC2 Without intron* | This study |
| HG3 | *MATa his3Δ200 trp1Δ63 leu2Δ0 met15Δ0 ura3Δ0 APE2 Without intron* | Agarwal and Ansari, 2016 |
| AA7 | *MATa his3Δ200 trp1Δ63 leu2Δ0 met15Δ0 ura3Δ0 ASC1 Without intron* | Agarwal and Ansari, 2016 |
| NEA6 | *MATa his3Δ200 trp1Δ63 leu2Δ0 met15Δ0 ura3Δ0 IMD4 Without intron* | Agarwal and Ansari, 2016 |
| HG2 | *MATa his3Δ200 trp1Δ63 leu2Δ0 met15Δ0 ura3Δ0 YPL109C Without intron* | This study |
| KMD9 | *MATa his3Δ200 trp1Δ63 leu2Δ0 met15Δ0 ura3Δ0 INO1::Act1 intron at 100 position* | This study |
| AMR2 | *MATa his3Δ200 trp1Δ63 leu2Δ0 met15Δ0 ura3Δ0 INO1::Act1 intron at 500 position* | This study |
| KMD10 | *MATa his3Δ200 trp1Δ63 leu2Δ0 met15Δ0 ura3Δ0 INO1::Act1 intron at 800 position* | This study |
| KMD11 | *MATa his3Δ200 trp1Δ63 leu2Δ0 met15Δ0 ura3Δ0 INO1::Act1 intron at 1400 position* | This study |
| KMD18 | *MATa his3Δ200 trp1Δ63 leu2Δ0 met15Δ0 ura3Δ0 INO1::Act1 intron at TGA+10 position* | This study |
| NEA11 | *MATa his3Δ200 trp1Δ63 leu2Δ0 met15Δ0 ura3Δ0 IMD4::Act1 intron at 461 position* | This study |
| AG1 | *MATa his3Δ200 trp1Δ63 leu2Δ0 met15Δ0 ura3Δ0 IMD4::Act1 intron at 750 position* | This study |
| KMD6 | *MATa his3Δ200 trp1Δ63 leu2Δ0 met15Δ0 ura3Δ0 IMD4::Act1 intron at TGA+20 position* | This study |
| KMD13 | *MATa his3Δ200 trp1Δ63 leu2Δ0 met15Δ0 ura3Δ0 IMD4 Without intron, SUA7-HA::HIS* | This study |
| AG7 | *MATa his3Δ200 trp1Δ63 leu2Δ0 met15Δ0 ura3Δ0 IMD4::Act1 intron at 461 position, SUA7-HA::HIS* | This study |
| AG5 | *MATa his3Δ200 trp1Δ63 leu2Δ0 met15Δ0 ura3Δ0 IMD4::Act1 intron at 750 position, SUA7-HA::HIS* | This study |
| KMD14 | *MATa his3Δ200 trp1Δ63 leu2Δ0 met15Δ0 ura3Δ0 IMD4::Act1 intron at TGA+20 position, SUA7-HA::HIS* | This study |

| | | |
|---|---|---|
| NEA7 | *MATa his3Δ200 trp1Δ63 leu2Δ0 met15Δ0 ura3Δ0 IMD4 Without intron, CCL1-TAP::URA3* | This study |
| SAM30 | *MATa his3Δ200 trp1Δ63 leu2Δ0 met15Δ0 ura3Δ0 IMD4::Act1 intron at 461 position, CCL1-TAP::URA3* | This study |
| AG6 | *MATa his3Δ200 trp1Δ63 leu2Δ0 met15Δ0 ura3Δ0 IMD4::Act1 intron at 750 position, CCL1-HA::HIS* | This study |
| KMD16 | *MATa his3Δ200 trp1Δ63 leu2Δ0 met15Δ0 ura3Δ0 IMD4::Act1 intron at TGA+20 position, CCL1-TAP::URA3* | This study |

**Table S2: Primers/probes used in RT-PCR, ChIP, CCC and TRO**

ChIP Primers

| **Primer Name:** | **Sequence:** |
|---|---|
| 5′ IMD4- P | ATGATAGACTTGAGGGACGCAC |
| 3′ IMD4- P4 | CAGCATCTCGAAGAGCAAAAA |
| 5′ IMD4- P2 | GCCATATAAATATCAGTTGAGAATCC |
| 3′ IMD4- P2 | GTATGTCTTCAAATGTTCTAAAGCC |
| 5′ IMD4- CCC | TGGATTACAAAAAGGCTTTAGAAC |
| 3′ IMD4- CCC | CAGCATCTCGAAGAGCAAAAA |
| 5′ IMD4- T | GATTCTGTGCATTTGAGGAGC |
| 3′ IMD4- T | GGTGTAAAGGGTTTAGATGAAGC |

YPL109 Primers

| **Primer Name:** | **Sequence:** |
|---|---|
| 5′ YPL109C- A1 | TTTTCATAAAATACCTATAAG |
| 3′ YPL109C- A1 | CTGAAATTTCTAAAAACCT |
| 5′ YPL109C- P1 | CTGTGAGGGTTTGATATTGATGAG |
| 3′ YPL109C- T1 | TGCATCTTTTATAATTGCGACTTCC |
| 5′ YPL109C- F1 | CCTCTTCTCCTATTATACCCCATAT |
| 3′ YPL109C- R1 | CGTTACTATGCAATTTACCCAAC |
| 5′ YPL109C- pRS | TATCAAACCCTCACAGAATAGAGATAAAGAACATCAGAACC ATCTGGGCAGCGGCATCAGAGCAGATTG |
| 3′ YPL109C- pRS | GCGAACACGTGGAAGTCGCAATTATAAAAGATGCATAAAAA GAAAGAATACTGTGCGGTATTTCACACCG |

INO1 Primers

| **Primer Name:** | **Sequence:** |
|---|---|
| 5′ INO-INT-up100 | CAAGTGCACGTACAAGGACAACGAGCTGCTCACCAAGTA CAGCTACGAAAGTATGTTCTAGCGCTTGCAC |

| | |
|---|---|
| 3′ INO-INT-down100 | AGTGGGCGTTACATCGAAGCGGCCACTAGCTGTCTTCGTAACTACAGCATCTAAACATATAATATAGCAACAAAAAGAATG |
| 5′ INO-INT-up500 | AACGCAGATCTATACGAAGCTATGCAGAGAAGTCAAGTTCTCGAATATGAGTATGTTCTAGCGCTTGCAC |
| 3′ INO-INT-down500 | AAGGAAGAGGCTTCACCAAGGACATCTTCGCCTTCAAGCGTTGTTGCAGACTAAACATATAATATAGCAACAAAAAGAATG |
| 5′ INO-INT-up800 | GTAGAAGTATCTCCTGGTGTTAATGACACCATGGAAAACCTCTTGCAGTCGTATGTTCTAGCGCTTGCAC |
| 3′ INO-INT-down800 | CTGCTGCAAAGATCGTGGAAGGAGCAATCTCTTCATGGTCATTCTTAATACTAAACATATAATATAGCAACAAAAAGAATG |
| 5′ INO-INT-up1400 | GTCATGACTGAGTTTTGTACAAGAGTGTCCTATAAGAAGGTGGACCCAGTGTATGTTCTAGCGCTTGCAC |
| 3′ INO-INT-down1400 | AGAAGGTTAAAACTGGATAAAAGTTCTCGAATTTGCCAGCATCTTCTTTACTAAACATATAATATAGCAACAAAAAGAATG |
| 5′ INO-INT-TGA+10 | GCCTTCTCAAAACGAACTAAGATTTGAAGAGAGATTGTTGTAATCTCATTGTATGTTC |
| 3′ INO-INT-TGA+10 | AATGTCTTGTTTTTTTATAGGTAGGCGGAAAAAGAAAAAGAGAGTCGTTGCTAAACA |
| 5′ INO1 A1 | GATATCCAGAATTTCAAAGAAGAAAAC |
| 3′ INO1 A2 | TATTCTGCGGTGAACCATTAATATAG |
| 5′ INO1 S1 | CAGAAAAGCTGATGAATGACTCTG |
| 3′ INO1 S2 | TTGATGGTTGGAGTTGTGACCG |
| 3′ 18S | GACGGAGTTTCACAAGATTACC |
| 3′ Oligo dT | TTTTTTTTTTTTTTTTTTTTTTTTT |
| 5′ 18S F | GGAATAATAGAATAGGACGTTTGG |
| 3′ 18S R | GTTAAGGTCTCGTTCGTTATCG |
| 5′ INO1 F2 | TGCTCACCAAGTACAG |
| 3′ INO1 R2 | GTAGTTTGGTTGCTTAACGCC |
| 5′ INO1 F3 | GGCGTTAAGCAACCAAACTAC |
| 3′ INO1 R3 | CCGACGGGCTTCATATATTTG |
| 5′ INO1 F4 | GGCGTTAAGCAACCAAACTAC |
| 3′ INO1 R4 | CCGACGGGCTTCATATATTTG |
| 5′ INO1 F5 | GTATTAAACCGGTCTCCATTGC |
| 3′ INO1 R5 | CAACAATCTCTCTTCGAATCTTAG |
| 5′ INO1 F6 | GTATTAAACCGGTCTCCATTGC |
| 3′ INO1 R6 | ATAGGTAGGCGGAAAAAG |

IMD4 Primers

| **Primer Name:** | **Sequence:** |
|---|---|
| 5′ IMD4 ACT1 461 | AGTTAAGGTTATGAAGAGAAAGTTTGGTTTCTCTGGCTTCCCAGTTACTGGTATGTTCTAGCGCTTGCAC |

| | |
|---|---|
| 3′ IMD4 ACT1 461 | ATCACGAGAGGTGACTAACCCAACTAACTTACCTGGACAC<br>TTACCGTCTTCTAAACATATAATATAGCAACAAAAAGAATG |
| 5′ IMD4 ACT1 750 | TTCTAAATCCGCCACCACCAAGCAATTGCTATGTGGTGCT<br>GCAATTGGTACTATCGAAGCTGATAAGGAAAGATTAAGAC |
| 3′ IMD4 ACT1 750 | TTTGATCATGTTCAATTGGAAAACAGAGTTACCTTGAGAG<br>GAATCTAAGATAACAACATCCAAACCTGCTTCGACTAATA |
| 5′ IMD4 ACT1 TGA+20 | CACTCCTATGAAAAACGTCTATACAATTGAGTAAGCATCCA<br>TAGATATTTGTATGTTCTAGCGCTTGCAC |
| 3′ IMD4 ACT1 TGA+20 | AGTTTTGTTGATCTCTATGAAGGTATATTTATATGCAAAAAT<br>AAACTTTTCTAAACATATAATATAGCAACAAAAAGAATG |
| 5′ IMD4- A1 | TGTTGTCAAGAGCGGATTTG |
| 3′ IMD4- A2 | AATCTGGGAAAGTTTCTTTAATCC |
| 3′ IMD4- UP1 | GATTCAAATCATGCTTGGCTC |
| 5′ IMD4- UP | CGCATTTTTTCATCTCTTTTTC |
| 3′ IMD4- UP | TTTGTTAGGTTTCTTCAAAGCTATG |
| 5′ IMD4- P1 | AGAGTTTTTCACATTAGGGCTGC |
| 3′ IMD4- T1 | CTTATTGAAGTATGTACAGTGGAAATAG |
| 5′ IMD4- F1 | TGGATTACAAAAAGGCTTTAGAAC |
| 3′ IMD4- R1 | CTTCAGTGACTGTGTCCATAGGAG |
| 5′ IMD4- J | TGGTATGTTAGCTGGTAC |
| 3′ IMD4- T | GGTGTAAAGGGTTTAGATGAA |
| 3′ IMD4- T2 | CGAACTGAAAAACGAAAATAAGAA |
| 3′ IMD4- T3 | CACAATTCAGGCGTGTTGC |
| 3′ IMD4- T4 | TCGCCTAAACATGCTTCC |
| 3′ IMD4- T5 | TGAAGATGAGCTGGAAAA |
| 5′ IMD4- ACT1 | CATGTACTAACATCGATTGCTTC |
| 5′ IMD4 FP | AGGAATTGATGGACTCCACAA |
| 3′ IMD4 RP | GGTTTGTTTTAGGATTTCATTACC |
| 5′ IMD4 FM | TGTTGTCAAGAGCGGATTTG |
| 3′ IMD4 RM | AATCTGGGAAAGTTTCTTTAATCC |
| 5′ IMD4 FT | TGGTATGTTAGCTGGTAC |
| 3′ IMD4 RT | AAGAATGATAGCAAATAGAGAAAGC |

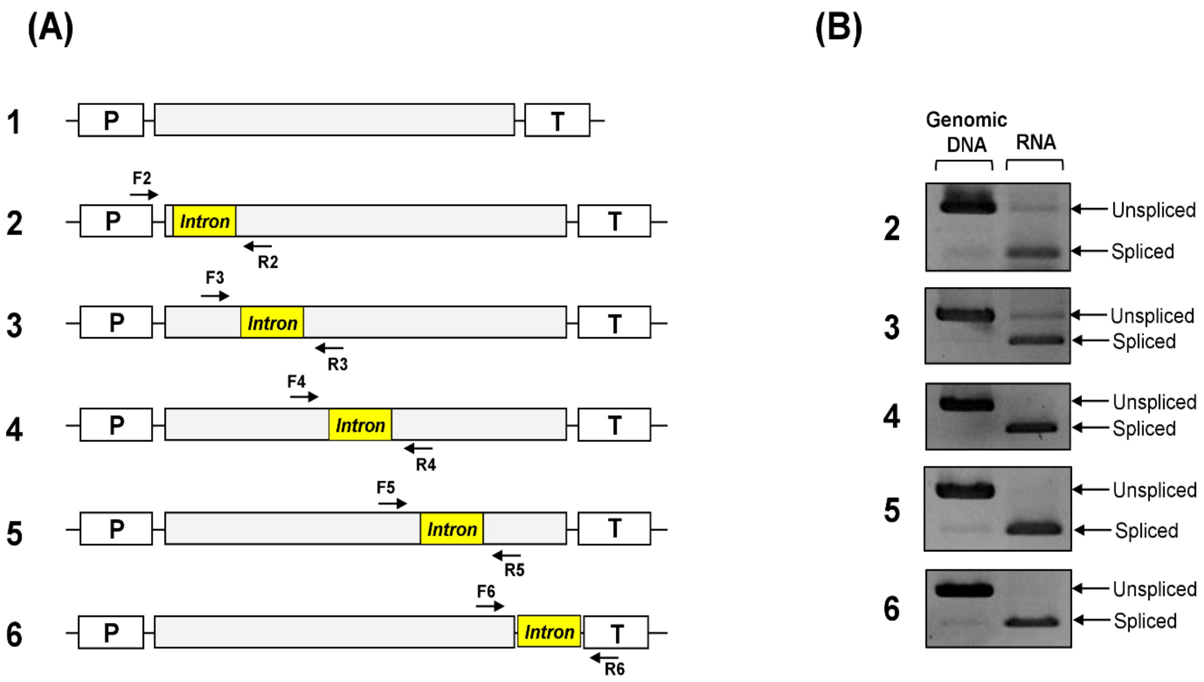


**Supplementary Figure 1.** The *ACT1* intron at different positions in the *INO1* gene is spliced efficiently. **(A)** Schematic depiction of *INO1* gene with the *ACT1* intron at five different positions within the gene. Arrows indicate the position of primers used in RT-PCR analyses. **(B)** RT-PCR analysis of *INO1* mRNA with the intron at five different positions. Genomic DNA PCR indicate the position of unspliced transcripts. The expected positions of spliced and unspliced transcripts obtained by RT-PCR approach for every construct is indicated.

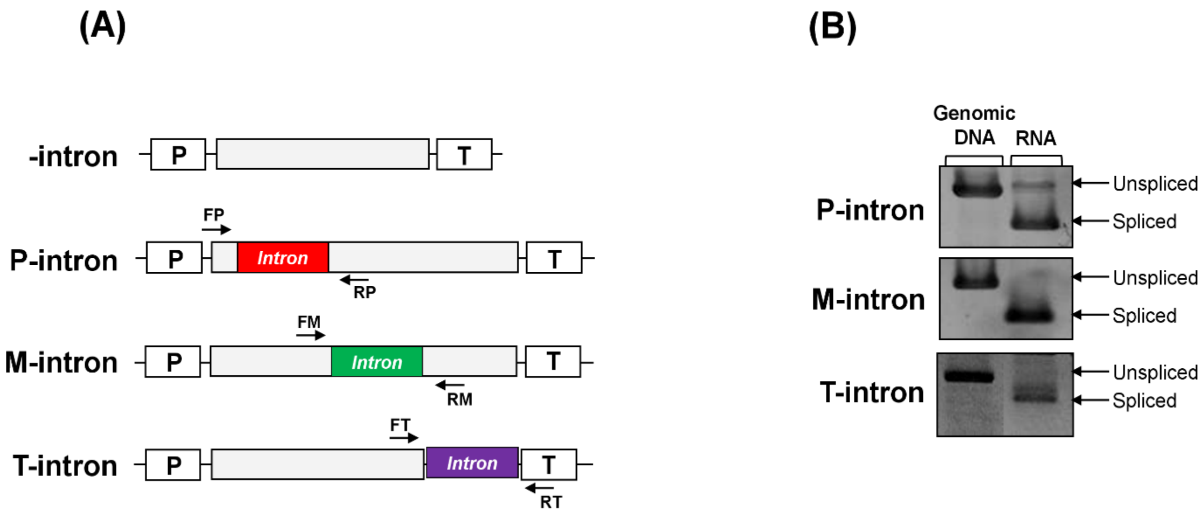


**Supplementary Figure 2.** The *ACT1* intron at different positions in the *IMD4* gene is spliced efficiently. **(A)** Schematic depiction of *IMD4* gene with the *ACT1* intron at three different positions within the gene. Arrows indicate the position of primers used in RT-PCR analyses. **(B)** RT-PCR analysis of *IMD4* mRNA with the intron at three different positions. Genomic DNA PCR indicate the position of unspliced transcripts. The expected positions of spliced and unspliced transcripts obtained by RT-PCR approach for every construct is indicated.
